# Functionalized Gold Nanoparticles as Biosensors for Monitoring Cellular Uptake and Localization in Normal and Tumor Prostatic Cells

**DOI:** 10.3390/bios8040087

**Published:** 2018-10-04

**Authors:** Marianna Pannico, Anna Calarco, Gianfranco Peluso, Pellegrino Musto

**Affiliations:** 1 Institute for Polymers, Composites and Biomaterials, National Research Council of Italy, 80078 Pozzuoli, Italy; 2 Institute of Agro-environmental and Forest Biology, National Research Council of Italy, 80131 Naples, Italy; anna.calarco@ibaf.cnr.it (A.C.); gianfranco.peluso@ibaf.cnr.it (G.P.)

**Keywords:** SERS, nanomaterials, biosensing, prostate cancer, single cell spectroscopy

## Abstract

In the present contribution the fabrication and characterization of functionalized gold nanospheres of uniform shape and controlled size is reported. These nano-objects are intended to be used as Surface Enhanced Raman Spectroscopy (SERS) sensors for *in-vitro* cellular uptake and localization. Thiophenol was used as molecular reporter and was bound to the Au surface by a chemisorption process in aqueous solution. The obtained colloidal solution was highly stable and no aggregation of the single nanospheres into larger clusters was observed. The nanoparticles were incubated in human prostatic cells with the aim of developing a robust, SERS-based method to differentiate normal and tumor cell lines. SERS imaging experiments showed that tumor cells uptake considerably larger amounts of nanoparticles in comparison to normal cells (up to 950% more); significant differences were also observed in the uptake kinetics. This largely different behaviour might be exploited in diagnostic and therapeutic applications.

## 1. Introduction

The advent of nanomaterials’ technology has opened up enormous opportunities in the bio-sensing area, allowing for the fabrication of constructs that are small enough to be targeted in the interior of single living cells and their intracellular compartments. Once in place, these nano-objects can serve several purposes: when decorated with suitable molecular reporters, they can be readily traced by different spectroscopic techniques, thus allowing their localization in the cellular environment [1,2,3,4,5,6]. Additionally, functionalized nanoparticles have been demonstrated for the cellular detection of early disease markers and as multifunctional substrates for targeted drug-delivery [7,8,9,10]. For example, chemotherapic treatments with nanoparticle carriers selectively targeting to disease cells and/or to specific subcellular structures (mitochondria or cell nuclei) hold great promise for improving treatment efficacy while reducing the side effects [7,8,9,11]. Numerous applications of nanotechnology in clinical practice have already emerged, giving rise to the contemporary, fast growing field of nanomedicine [8,9,10,11]. To reach the sensitivity levels that enable localization in the cellular environment, most nano-objects used for bio-sensing contain a core-element providing an intense electromagnetic signal that can be detected by non-invasive and non-ionizing spectroscopies. Among the different materials that have been proposed (polymers, inorganic oxides, transition metals, and related oxides) the noble metals silver and gold exhibit distinct advantages. With these elements, nanofabrication of objects of various geometries, from simple spherical shapes to complex multilayer architectures, has been achieved through the well-developed methodology of seed-growth. A fine control of size and size-distribution is possible by properly selecting the particle growing conditions [12,13]. Furthermore, the Ag and Au metal surfaces are highly responsive toward adsorption of molecular probes by both physisorption (electrostatic interactions) and chemisorption mechanisms (high reactivity to different functional groups, notably thiols, S—H), which facilitates surface functionalization. However, the principal advantage of Ag/Au based nanostructures relies on their strong, stable, and homogeneous plasmon resonance in a wavelength region comprising most of the available laser sources (i.e., 400–900 nm) [6,14,15]. This effect is exploited in Surface Enhanced Raman Spectroscopy (SERS), a molecular detection method that provides high sensitivity and specificity. Incident radiation hitting on a suitable metallic surface excites the surface plasmons, i.e., the quanta associated to the longitudinal waves propagating through the motion of the conduction electrons, generating enormous enhancement of the laser field, which results in an increase of the Raman signal typically in the range of 10^5^–10^9^ times with respect to spontaneous emission. To take full advantage of the plasmonic effect the analyte must be in contact with the metal surface or at least be very close to it because the field-enhancement decays exponentially with distance. Typically, the maximum gap from the sensing surface has to be ≈10 nm, and this is a primary consideration in the design of a SERS-based sensor. SERS spectroscopy has enormous potential for analytical applications provided that the very many variables that were involved in the complex plasmon-resonance process are suitably optimized [16,17].

Fluorescent probes have been employed with great success in biomedicine; however, a number of shortcuts prompted researchers in the bioanalytical chemistry area to explore alternative approaches. In particular, fluorophores display specific excitation profiles, which prevents multiplexed detection. The stability of these reporters can be compromised by photo-bleaching and chemical degradation, especially in the complex cellular environment. Fluorescence signals are generally broad and featureless and they may produce overlapped, ill-resolved bands that can be difficult to use for identification and quantification purposes [18,19]. Conversely, SERS substrates are robust and produce a spectroscopic fingerprint readily discernible in a complex mixture. The SERS spectrum is a highly specific pattern of signals with very narrow bandwidths (typically, less than 1 nm against over 50 nm in fluorescence spectra). This property provides the potential for multiplexed analyte detection [1,5,16,17] A further advantage of the SERS spectrum is that it is essentially unaffected by factors, like the presence of water, oxygen, and temperature, thus making it an ideal candidate to probe the cellular environment at the molecular level [3,5] Current research efforts are devoted to optimize nanofabrication and surface functionalization of the nanoobjects, and to develop advanced tools for surface characterization. A very promising approach, in this context, is the surface activation of gold/silver colloids with DNA/RNA aptamers, that, once secured on the metallic surface by covalent binding, allow for an exceptional selectivity towards target proteins/receptors coupled with the exquisite sensitivity of the plasmonic response [20,21,22,23,24]. Cell uptake demands an accurate control over the factors that regulate this process, such as geometry, size, and size distribution of the nanoobjects. In particular, for this application it is mandatory to avoid nanoparticle aggregation that can be tolerated, or even desirable in solution, wherein clustering produces a large number of hot-spots at the interparticle contact-points, which greatly enhance SERS signaling and the resulting sensitivity. Conversely, even the aggregates of few nanoparticles loose the capability of entering the cell body through the cellular membrane. It is equally important to develop robust probes that will retain their structural integrity in the presence of the biomolecules, enzymes and proteins present in the cellular environment. With this in mind, the present study reports on the fabrication of gold nanospheres to be used as *in-vitro* SERS probes to accurately quantify their uptake and localization in the cell interior. The goal was the development of a SERS-based method to consistently differentiate normal and tumor cell lines. In this context, the single-cell approach is intended to serve as a model system for further developments of the technique in clinical applications [25]. The particle shape and size were selected for promoting cell access, in the light of previous literature reports on human prostatic lines (PC3) [26]. Attention was paid to achieve shapes as homogeneous as possible, narrow size distributions, and to avoid particle aggregation. After functionalization with thiophenol (TP) as the SERS label, the probes were fully characterized by Transmission Electron Microscopy, image analysis, UV, and Raman spectroscopy. By monitoring cellular uptake as a function of incubation time, selective interactions of the SERS probes with normal and malignant lines of human prostatic cells were demonstrated. This largely different behavior might have relevant implications from both a diagnostic and a therapeutic perspective.

## 2. Experimental Section

### 2.1. Materials

Trisodium citrate (TC), gold(III) chloride trihydrate (HAuCl_4_·3H_2_O), ascorbic acid, sodium borohydride (NaBH_4_), Cetyltrimethylammonium bromide (CTAB), thiophenol (TP), and ethanol were purchased from Sigma-Aldich and were used as received. Milli-Q water was used for the preparation of gold nanoparticles.

### 2.2. Synthesis of Gold Nanoparticles

The gold nanoparticles (AuNPs) were prepared by the seeding growth method [12,13], which is detailed below:

#### 2.2.1. Preparation of Gold Seeds Solution

The gold seeds (~ 3 nm) solution was obtained at room temperature by sodium borohydride reduction of the gold salt in the presence of citrate as capping agent. A 20 mL aqueous solution containing the same molar concentration (2.5 × 10^−4^ M) of HAuCl_4_ and trisodium citrate was prepared in a glass bottle. Next, 600 μL of ice-cold freshly prepared 0.1 M NaBH_4_ aqueous solution was added to the solution while gentle stirring. After the addition of NaBH_4_ the solution immediately turned pink, indicating seed formation. The seed solution was left undisturbed at room temperature for 3 h and it was then used for particles growth.

#### 2.2.2. Preparation of Growth Solution

A 20 mL aqueous solution of 2.5 × 10^−3^ M HAuCl_4_ was prepared in a glass bottle. Next, CTAB (final concentration 0.08 M) was added to the solution under stirring and the mixture was heated until it turned clear orange. The growth solution was cooled down to room temperature before use.

#### 2.2.3. Seeding Growth

Under gentle stirring, 1 mL of freshly prepared ascorbic acid aqueous solution (0.1 M) was added to 18 mL of the growth solution. While stirring, the seed solution was added to the mixture, which immediately turned wine red, confirming the formation of gold nanoparticles. The colloidal solution was stirred for 10 min and then centrifuged three times at 13,000 rpm, 35 °C for 15 min. At least three centrifugation cycles were necessary to purify the AuNPs solution since free CTAB molecules are quite toxic to cells at submicromolar doses. Complete removal of free CTAB was confirmed by reflection FTIR spectroscopy on the supernatant solution. Alkilany et al. [27,28] demonstrated that the cytotoxicity of gold nanoparticles prepared using CTAB is to be entirely attributed to free CTAB molecules in solution and not to the CTAB molecules that were bound on the surface of the nanoparticles. The pH of the pristine colloid (prior to centrifugation) was 2.80 and it was changed to 4.90 after centrifugation and re-dispersion in water. The pH change did not induce nanoparticle aggregation, as demonstrated in the forthcoming discussion.

#### 2.2.4. Functionalization of Gold Nanoparticles

After centrifugation and re-dispersion in water, the gold nanoparticles were functionalized with thiophenol (TP) by adding a 100 μM ethanol solution of TP to the AuNPs colloidal solution (*v*/*v* 40:60), gently stirring for 10 min at room temperature. TP has a very strong affinity to the gold surface through the thiol group (-SH) that facilitate its exchange with CTAB on the nanoparticles surface. It was demonstrated that the adsorption behavior of TP on gold nanoparticles results in the formation of a gold phenylthiolate (C_6_H_5_SAu) surface complex with the formation of a self-assembled monolayer [29]. The TP functionalized nanoparticles (AuNPs/TP) were further centrifuged at 13,000 rpm, 35 °C for 25 min to remove the excess TP. The purified AuNPs/TP were finally used for the incubation step with cells.

### 2.3. Cell Culture and Cytotoxicity Assay

The human prostate adenocarcinoma cell lines (PC3) and the immortalized non-cancerous prostate epithelial cell line (PNT2) were purchased from the European Collection of Cell Cultures (ECACC, Salisbury, UK) and tested for mycoplasma contamination. Cells were maintained in RPMI 1640 supplemented with 10% fetal bovine serum (FBS), 1% L-glutamine, 50 U/mL penicillin, and 50 mg/mL streptomycin (growth medium; all from GIBCO, Milan, Italy) at 37 °C in a humidified atmosphere containing 5% CO_2_.

Before cells incubation, the AuNPs/TP cytotoxicity was verified according to the procedure shown below. AuNPs/TP cytotoxicity was evaluated on cancer and normal cell lines by 3-(4,5-dimethylthiazol-2-yl)-2,5-diphenyltetrazolium bromide (MTT, Roche Holding AG, Milan, Italy) as reported previously [30]. MTT cell proliferation assay measured the enzymatic cleavage of the tetrazolium reagent WST-1 to colored formazan in the presence of mitochondrial dehydrogenases from living cells. Cells (5000 cells per well) were seeded within 200 μL growth media in 96-well plates one day before incubation with a wide range of AuNPs/TP concentrations (0 to 2.3 nM) for 6, 12, 24, 48, and 72 h. Untreated cells were considered as the control. Following incubation, the solutions were replaced with 200 μL fresh growth medium, and 20 μL MTT (5 mg/mL in PBS), and were incubated at 37 °C for 4 h. The purple formazan products were then dissolved with 100 μL DMSO. The absorbance was measured at 570 nm by means of a microplate reader (Cytation, Milan, Italy). The cytotoxicity results were expressed as the percentage of viable cells with respect to the untreated control cells. For every single measurement, six wells from the 96-well plate were exposed to the same AuNPs concentration, and each measurement was performed in triplicate. The biocompatibility of AuNPs was further confirmed with a LDH assay kit (Roche, Roche Holding AG, Milan, Italy), as reported in ref. [30]. LDH assay is based on the measurement of lactate dehydrogenase (LDH) released into the growth media when the integrity of the cell membrane is lost. As a positive control, the cells were completely lysed with Triton X-100, according to the manufacturer’s instructions. LDH activity was reported as a percentage respect to the control.

### 2.4. Nanoparticles Uptake

The day before the experiments, cells were seeded at a density of 5 × 10^5^ on a support optimized for Raman and visible microscopy inspection (MirrIR, *low-e* microscope slides from Kevley Technologies, Chesterland, Ohio, USA). Then, the growth medium was replaced with fresh serum free medium (pH = 7–7.2) containing 0.2 nM AuNPs/TP. The cells were rinsed twice with PBS prior to be fixed in 4% paraformaldehyde for 10 min (4 °C). Prior to Raman analysis, the fixed cells were rinsed with water. The uptake experiments were repeated three times with different cells preparations and AuNPs batches. For each incubation time, 10 isolated cells were mapped. The results shown hereafter are therefore, averaged over 30 PNT2 and 30 PC3 cells for each uptake time.

### 2.5. Techniques

#### 2.5.1. Transmission Electron Microscopy (TEM)

The size and shape of gold nanoparticles were examined by bright field transmission electron microscopy analysis that was performed on a FEI Tecnai G12 Spirit Twin (LaB6 source, Eindhoven, The Netherlands) equipped with a FEI Eagle 4 K CCD camera (Eindhoven, The Netherlands) operating with an acceleration voltage of 120 kV. Samples for TEM examination were prepared by th deposition of the AuNPs on a carbon-coated copper grid, achieved by immersing the grid in the colloidal solution, followed by drying. At least 20 TEM images were acquired in different sample areas and they were used for the Statistical Image Analysis (SIA).

#### 2.5.2. Statistical Image Analysis (SIA)

**Figure 1:**
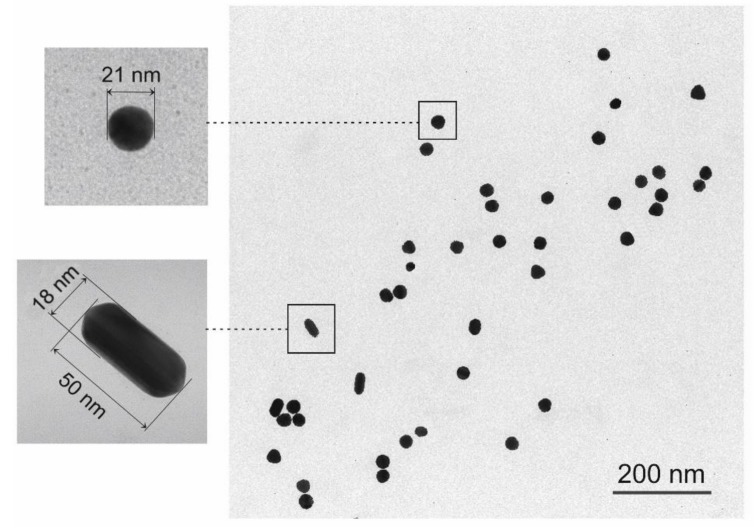
Typical Transmission Electron Microscopy (TEM) micrograph of gold nanoparticles. The insets are the high-magnification TEM of the indicated gold nanoparticles (AuNPs).

Particle analysis of an image dataset object was accomplished in two steps: the first consists in obtaining a binary image where pixel values are either 0 or 1 where 0 represents non-particle pixels and 1 represents potential particle pixels. This is made by specifying a threshold level where pixels having a value below or above the threshold are assigned value 0 or 1, respectively. In the present case, the threshold value is automatically determined within the analysis (auto thresholding method). The second step consists in calculating the properties of each particle region. Properties include area, perimeter, centroid coordinates, shape properties (circularity, aspect ratio, roundness, and solidity), and Feret’s diameters. Finally, statistics on the above parameters are evaluated over the whole particle population, providing mean, median, minimum, maximum, and standard deviation. The analysis is performed on the TEM images transferred to an off-line computational facility in the form of standard .tif file format. The analysis provides very reproducible and accurate results, owing to the sharp contrast of the images wherein the particles appears as black objects over a white background (see Figure 1). Use was made of the PLS/MIA toolbox, from Eigenvector Research Inc., Manson, WA, USA, running within the MATLAB computational platform (Mathworks, Natick, MA, USA).

#### 2.5.3. UV-VIS Spectroscopy

Absorption spectra of the AuNPs colloids were measured by UV spectrophotometer with single monochromator (V−570 from Jasco, Easton, USA) using 1.00 cm quartz cells with a scan speed of 400 nm/min in the wavelength range from 300 to 800 nm. UV-VIS spectroscopy was used for determining the AuNPs molar concentration and for verifying the stability of the colloidal solutions before and after functionalization with TP.

#### 2.5.4. Dynamic Light Scattering (DLS) and Zeta-Potential Measurements

DLS and Zeta potential of the CTAB-coated nanoparticles were measured with a Malvern Zetasizer nano series (Malvern Instruments Ltd., Malvern, UK). A standard UV/VIS quartz cuvette was used for DLS, while for Z-potential, a capillary cell type ZEN 1020 was employed. DLS and Zeta-potential measurements were done before and after the centrifugation cycles to verify the AuNPs solution stability. The reported values are the estimated mean of three measurements.

#### 2.5.5. Raman Spectroscopy

The Raman spectra were collected by a confocal Raman spectrometer (Labspec Aramis, from Horiba-Jobin Yvon, Edison, NJ, USA) operating with a 632 nm diode laser as the exciting source. The 180° back-scattered radiation was collected by an Olympus metallurgical objective (MPlan 50×, NA = 0.75 or 10×, NA = 0.25) with confocal and slit apertures set to 300 μm; a grating with 600 grooves/mm was used throughout. The radiation was focused onto a CCD detector (Synapse Mod. 354308) cooled at −70 °C by a Peltier module. The laser power that was measured at the output of the objective was 1.87 mW, which resulted in a power density of 1.9 mW/μm^2^. A quartz cuvette with chamber volume of 700 μL, from Hellma GmbH & Co, Jena, Germany, was used to collect the spontaneous Raman spectrum of TP (liquid) and the SERS spectra of the functionalized AuNPs solution; both spectra were collected with the 10×/0.25 objective and 3 s of acquisition time. The SERS measurements on the cells after nanoparticles incubation were carried out in mapping mode: the quartz microscope slides with the seeded cells were accommodated on a piezo-electrically driven microscope stage with a spatial *x,y* resolution of 10 ± 0.5 nm and a *z* resolution of 15 ± 1 nm, which was scanned at a constant stage speed in the *x-y* plane with a 2.0 μm step size. The Raman maps on isolated cells were collected with 3 s exposure time using the 50×/0.75 objective. All of the collected Raman data were converted into ASCII format and transferred to the MATLAB computational platform for further processing. The Raman images were elaborated by in-house written codes, making use of the image-processing facilities of the MATLAB environment.

## 3. Results and Discussion

### 3.1. Nanoparticle Characterization

#### 3.1.1. TEM/SIA

**Figure 2:**
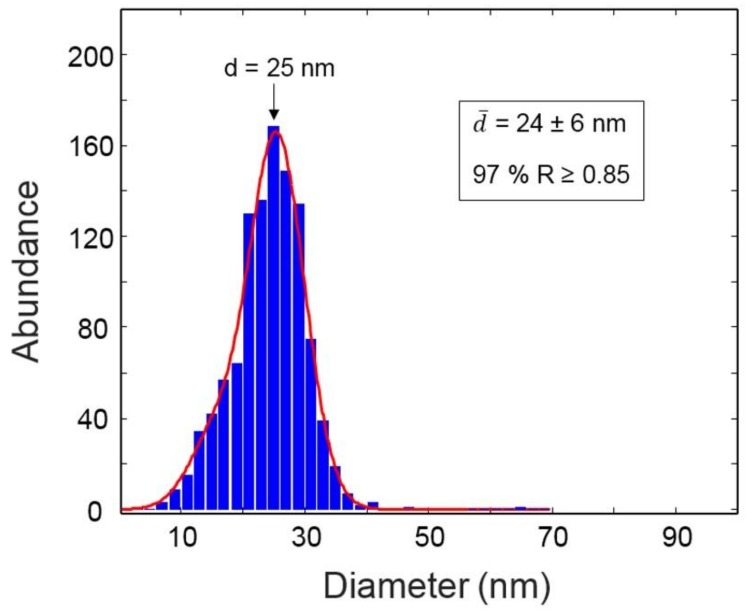
Size distribution of AuNPs.

Statistical Image Analysis (SIA) was performed on 20 TEM micrographs each containing from 80 to 120 nanoparticles, which provided a total population of ≅ 2000 units. The micrographs (see Figure 1) were obtained by deposition of the colloidal solutions on a TEM grid, followed by drying. The Roundness parameter, which is defined as:

(1) $$R=\frac{4\times A}{\pi \times d_{max}^{2}}\;$$

where, *A* is the particle area and *d_max_* is the major axis, was used to verify shape and shape homogeneity of the AuNPs. In Figure 2 is represented the distribution of particle sizes: 97% of the population is characterized by a spherical shape (roundness ≥ 0.85) and has an average diameter of 24 nm. The size distribution is narrow (Full width at Half maximum, FWHM, = 10.1 nm) and it has a pseudo-gaussian shape with some evidence of bimodal distribution in the form of a shoulder of the main component in the lower side of the histogram. A small fraction of nanoparticles with *R* ≤ 0.6 (less than 3% of the total population) takes the form of nanorods (see inset of Figure 1). In the whole set of TEM images, no evidence was found of particle aggregation, which is a pre-requisite to apply these constructs in cellular uptake studies.

#### 3.1.2. DLS and Z-Potential Measurements

The particles size distributions of the AuNPs colloid, as monitored by the DLS technique, is reported in Figure S1, Supplementary data, which gives a mean diameter (*z*-average value) of 27.6 ± 10.8 nm, in excellent agreement with the TEM/SIA results. DLS confirms a unimodal, relatively sharp size-distribution and the absence of aggregation phenomena.

The zeta potential measurements on AuNPs confirmed that the CTAB surfactant adsorbs onto the metal surfaces, forming a self-assembling bilayer in which the cationic trimethylammonium head groups of two adjacent CTAB molecules alternatingly face the metal surface and the solvent medium [31]. This arrangement makes the AuNPs surface positively charged; accordingly, the estimated zeta-potential of our AuNPs is +43 ± 3 mV. This value hardly changed, even after centrifugation and re-dispersion in milli-Q water (+40 ± 2 mV), confirming the stability of the bilayer structure [30,32]. On functionalization, TP substitutes, at least partially, the CTAB bilayer owing to the higher reactivity of the Au surface towards the thiol group compared to the electrostatic interaction formed with the ammonium cation. This is confirmed by the conspicuous drop of zeta potential after functionalization (+16 ± 0.3 mV). In terms of aggregation, the CTAB-containing nanoparticles are expected to have the highest stability because, due to the external positive charge, they repel each other. However, also, the TP functionalized AuNPs are expected to remain isolated, owing to of the neutral character of the external TP layer. This point will be further discussed in the forthcoming paragraph on UV spectroscopy measurements.

#### 3.1.3. UV-VIS Absorption Spectroscopy

**Figure 3:**
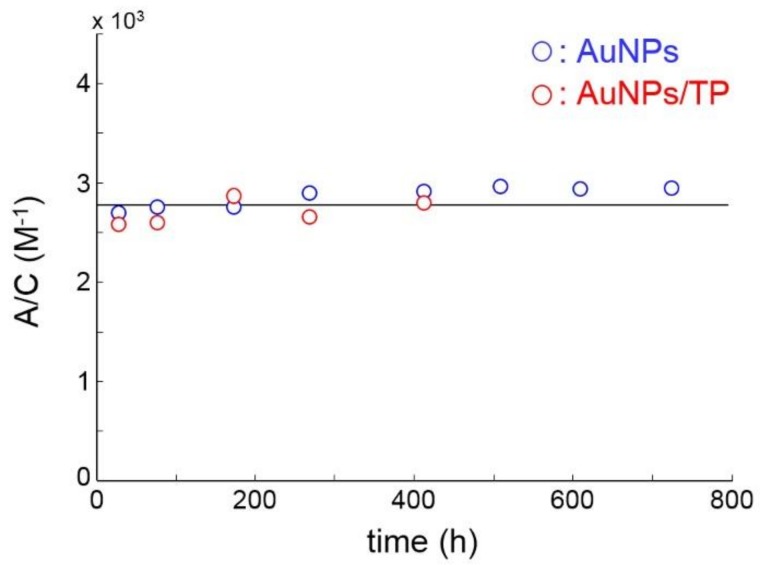
Concentration-normalized Absorbance of AuNPs (blue circles) and AuNPs/TP (red circles) as a function of ageing time.

The colloids were further characterized by UV-Vis spectroscopy, which provides information on nanoparticle concentration and on the occurrence of aggregation phenomena [14,33]. The collected UV spectra are reported in the inset of Figure S2, Supplementary data. The localized surface plasmon resonance (LSPR) band is clearly visible as a peak centered at 526 nm. This position is characteristic of nanoparticles with an average dimension of 20 nm, while the shape is indicative of a narrow, unimodal distribution [34,35], which is in full agreement with TEM/SIA and DSL results. The absorbance vs. concentration plot (see Figure S2, Supplementary data) displays a Beer-Lambert behaviour (i.e., it is linear through the origin, correlation coefficient, R^2^ = 0.999), which affords an accurate quantitative analysis of the Au amount in the batches used for cell-uptake experiments. This information, coupled with the geometrical parameters from TEM, provide the AuNPs concentration (vide infra). The set of standards was obtained by the successive dilution of an original solution; absolute values of Au concentration (mol/L) in the standards were estimated while assuming complete reduction of Au(III) to Au(0) in the initial batch. It is known that nanoparticles aggregation causes a red-shift of the LSPR band, with the formation of a second band at higher wavelengths generally appearing as a shoulder of the main component. Thus, as aggregation proceeds, the main SPR band lessens, and the shoulder concurrently increases. An isosbestic point is generally observed at a wavelength, depending on particle shape and aggregate size. The effect of aggregation on the maximum of the main band is weaker, due to the large difference between the positions of the two components [14]. In this light, we analyzed the LSPR height as a function of ageing time for both the pristine and the TP-functionalized AuNPs. The results, as represented in Figure 3, demonstrate the stability of both colloids over a long time period (up to 30 days for AuNPs; for AuNPs/TP the test was stopped after 17 days, since the nanoparticles were used within few hours for the cell uptake experiments). The results in Figure 3 and the UV spectra in Figure S3, Supplementary data, also demonstrate that substitution of the CTAB bilayer with the TP SAM does not affect the plasmonic response of the gold surface neither in terms of resonance efficiency (UV band intensity), nor in terms of SPR position (band maximum).

#### 3.1.4. SERS

**Figure 4:**
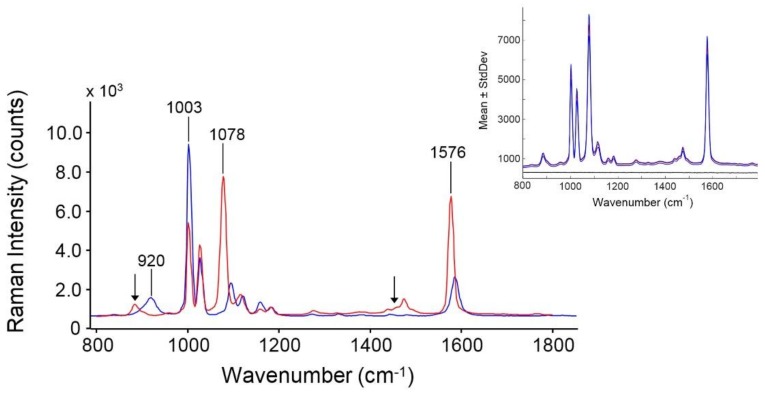
Blue trace: Raman spectrum of TP (liquid); Red trace: SERS spectrum of the AuNPs/TP colloid (30 μM in TP). The inset represents the SERS spectrum of AuNPs/TP colloid averaged over 20 points (red trace) ± the standard deviation (blue traces). The black trace in the inset represents the SERS spectrum of the native AuNPs.

The SERS spectrum of the AuNPs/TP colloid is compared in Figure 4 to the spontaneous Raman spectrum of liquid TP. The spectrum of the probe adsorbed onto the Au surface resembles, but is not coincident with, the reference spectrum (compare red and blue traces). In particular, the bands denoted by arrows belong to ethanol added to the colloidal solution to promote the solubilization of TP. The 920 cm^−1^ Raman band, attributed to C-S-H bending in TP [29], completely disappears in the SERS trace, which confirms the chemisorption of the probe with cleavage of the S-H bond and formation of a gold thiophenylate adduct, C_6_H_5_SAu. Two SERS peaks at 1078 and 1576 cm^−1^ increase appreciably with respect to their Raman counterparts, while moving at lower frequencies by − 12 and − 6 cm^−1^, respectively. Conversely, the SERS peak at 1002 cm^−1^ is weaker than the respective Raman signal. TP has been the subject of numerous theoretical and experimental investigations that provided reliable assignments for both the spontaneous and the SERS spectra [29]. These are summarized in Table 1, wherein the symmetry of the relative normal modes is also reported, assuming the chemisorbed TP molecules to adopt a C_2v_ point group.

The 1078 and 1576 cm^−1^ peaks (C—C symmetric and anti-symmetric stretching, respectively) both belong to the b_1_ symmetry species, which, according to standard group theory arguments [36], have the polarizability component entirely localized on the *z* axis (only α_zz_ ≠ 0). According to the SERS selection rules, the intensity enhancement of the b_1_ peaks denotes that the molecular axis (i.e., the principal symmetry axis) is oriented almost perpendicular to the metal surface. Conversely, the 1002 cm^−1^ peak belongs to the a_1_ symmetry species, for which the polarizability tensor has finite values for all of the three diagonal terms (α_xx_, α_yy_, and α_zz_ ≠ 0). The marked lowering of SERS intensity indicates that for this normal mode the two α_xx/_α_yy_ components strongly prevail over α_zz_. The above considerations are in agreement with earlier literature results [29].

To evaluate the SERS performances of the nanoparticles, SERS spectra were collected at different points in the AuNPs/TP colloid. The absolute enhancement factor, *EF*, was calculated as:

(2) $$\;EF=\frac{I_{SERS}}{I_{REF}}\cdot \frac{N_{REF}}{N_{SERS}}\;$$

where *I_SERS_* is the integrated area of a specific SERS signal (at 1576 cm^−1^) and *I_REF_* is the integrated area of the corresponding Raman signal (at 1587 cm^−1^), both normalized for exposure time.

Analogously, *N_SERS_* and *N_REF_* represent the number of molecules contributing to the SERS and the Raman signal, respectively. Both *N_SERS_* and *N_REF_* depend on sampling volume, which, in the present case, is invariant, because the same collection parameters were adopted in both experiments. *N_REF_/l* was obtained from the molar concentration of the reference TP/EtOH solution (10 wt %). To get *N_SERS_* we need to know the total available gold surface, which was evaluated from the Au concentration (by UV) and from the mean diameter of the spherical nanoparticles (by TEM). Thus, the volume, *V_NP,_* of a single nanoparticle of radius *r_NP_* $\left(V_{NP}=\frac{4}{3}\pi r_{NP}^{3}\right)$ is equal to 7.24 × 10^−18^ cm^−3^. The mass of a single nanoparticle, *m_NP_* = 1.40 × 10^−16^ g, was evaluated as:

(3) $$\;m_{NP}=\;V_{NP}\cdot D_{Au}\;$$

where *D_Au_* is the density of gold (19.32 g/cm^3^).

The mass of gold per unit volume in the colloid solution was divided by *m_NP_* to get the number of nanoparticles per unit volume, $N_{NP}\cdot l^{-1}$. The surface of a single nanoparticle, $S_{NP}=4\pi r_{NP}^{2}$, is 1.81 × 10^−11^ cm^2^, from which the total available gold surface per unit volume is: $S_{T}=S_{NP}\cdot N_{NP}$ = 3.8 × 10^4^ cm^2^ *l*^−1^.

The equatorial cross-section of the TP molecule, ρ, was evaluated by inspection of the Van der Waals surface of the structure relaxed at DFT/B3LYP level of theory (details in the data ), assuming an elliptical shape at the equator; ρ resulted to be 5.7 × 10^−15^ cm^2^. Therefore, the TP molecules bound onto a single nanoparticle, assuming that the formation of a self-assembled monolayer with normal *z-*axis orientation, is equal to $S_{NP}/\rho$ = 3175, and the bound TP molecules per unit volume (*l*) in the colloid is $S_{T}/\rho =6.63\;\times 10^{18}$. This figure corresponds to *N_SERS_/l*, the number of SERS active molecules per unit volume. The *EF* value from Equation (2) is, therefore, 1.5 × 10^6^, which is appropriate for sensitive SERS detection in cellular uptake studies [4,5,35]. The spectrum is also highly reproducible, as demonstrated by the low values of standard deviation for repeated measurements on different collection points (see inset of Figure 4).

### 3.2. Nanoparticles Cytotoxicity

To select a safe concentration of AuNPs for the cellular uptake studies, the influence of AuNPs concentrations on cellular cytotoxicity was investigated by applying the MTT and the LDH assays in parallel. The toxicity assays were carefully optimized to avoid the potential nanoparticle interferences.

**Figure 5:**
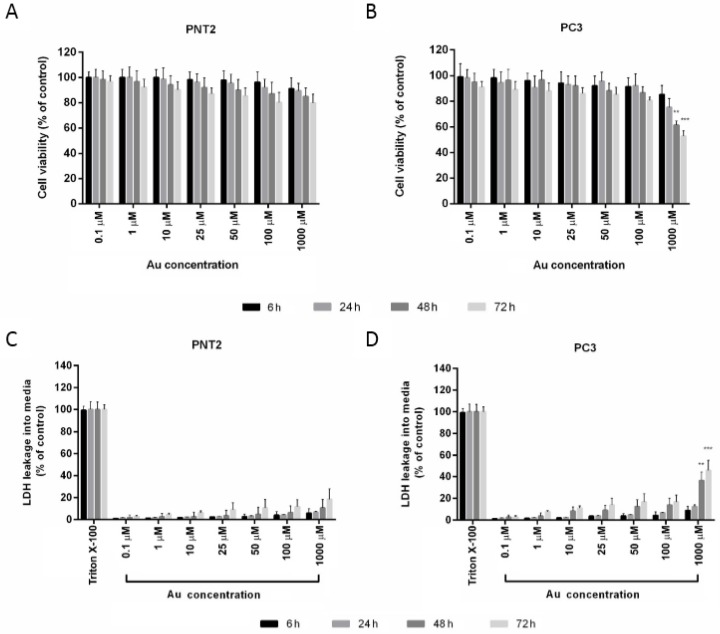
Cytotoxicity evaluation of AuNPs assessed by 3-(4,5-dimethylthiazol-2-yl)-2,5-diphenyltetrazolium bromide (MTT) and lactate dehydrogenase (LDH) assays in PNT2 (**A**,**C**) and PC3 (**B**,**D**) cells. Cells were incubated with increasing concentrations of Au from 0.1 to 1000 μM (corresponding to 0–2.3 nM in nanoparticles) for 6, 24, 48, and 72 h. In each experiment, four replicates per concentration were tested. The experiment was repeated three times. The data are mean values of three independent experiments (± SEM).

As shown in Figure 5, the PNT2 cell viability (Figure 5A) was unaffected by all tested AuNPs concentrations, even after 72 h of treatment, while the metabolic activity of PC3 cells (Figure 5B) decreased in a time- and concentration-dependent manner. In particular, the higher AuNPs concentration (2.3 nM, 211 μg/mL) displays a mild inhibitory effect on PC3 cells viability after 48 and 72 h (*p* < 0.01 and *p* < 0.001, respectively). In addition, LDH release levels indicated a slightly cellular membrane damage in PC3 cells (Figure 5D). Based on cytotoxicity assays, 0.2 nM (20 μg/mL) was selected for the uptake experiments.

Experimental observation has demonstrated that Thiol passivated AuNPs are stable for a long time, in sharp contrast with non-passivated particles [37]. In addition, recent papers have shown that hydrophobic NPs reveal attractive uptake kinetic in cancer cells. Indeed, the elevated cytotoxicity of docetaxel-loaded hydrophobic NPs (Dtxl-8P4 NPs) against tumor cells could be ascribed to the rapid cellular uptake and effective lysosomal escape [38]. However, the lower toxicity of Dtxl-8P4 NPs towards non-cancer cells has not been clearly explained. Here, we demonstrate that a possible explanation of this biological effect might be attributed to the poor uptake of hydrophobic NPs in normal cells. It can be envisaged that NPs systems might be designed, which could respond to changes in the surface biochemistry at cancer cell surfaces, in addition to an intracellular response.

### 3.3. Cellular Uptake

We have investigated the AuNPs/TP uptake in normal and tumor lines of human prostatic cells by SERS spectroscopy. TP was used as molecular reporter, because of its optimum SERS activity and the stability of the relative colloids. The neutral character of the ligand should not be detrimental for the present application. In fact, the uptake of neutral nanoparticles in several cell lines has been clearly detected in numerous studies [39,40,41]. The same batch of AuNPs/TP was used for the incubation with both PNT2 and PC3 cells for different incubation times (2, 4, and 6 h). The maximum incubation time was chosen according to previous literature reports, indicating a plateau of cellular internalization for gold nanoparticles after 6 h [42]. The uptake and localization into PNT2 and PC3 cells was monitored by Raman imaging experiments. Hereafter, we will report only a representative selection of the SERS maps showing the progression of AuNPs/TP uptake over time. The Raman images (color maps) were reconstructed by considering the peak intensity at 1078 cm^−1^ (C–C antisymmetric stretching). To quantify cell uptake, we evaluated the parameter *I_1078_*, i.e., the intensity of the analytical signal in the sum-spectrum, which was obtained by adding all SERS spectra that were collected within the cell body. This value is proportional to the number of internalized nanoparticles, provided that instrumental parameters are kept constant for all uptake experiments.

**Figure 6:**
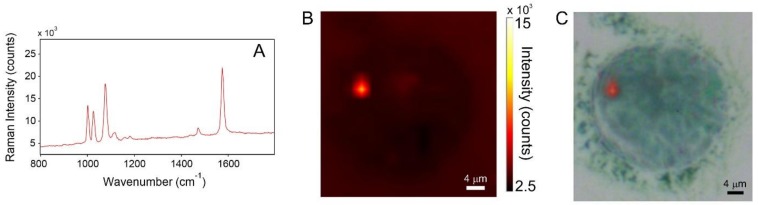
(**A**) SERS spectrum collected at the point of maximum SERS activity (red spot in the Raman image). (**B**) SERS intensity map of a PNT2 cell incubated for 2 h with AuNPs/TP. (**C**) superposition of the visible and SERS images.

**Figure 7:**
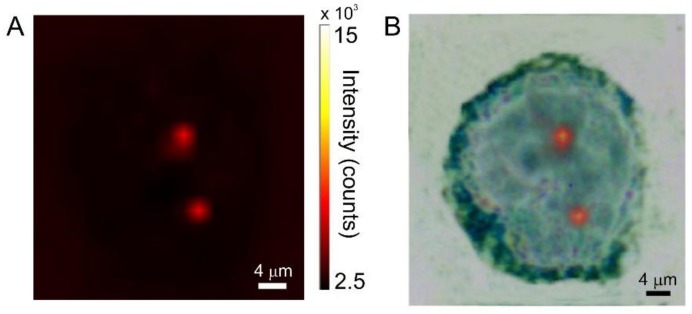
(**A**) SERS intensity map of a PNT2 cell incubated for 6 h with AuNPs/TP. (**B**) superposition of the visible and SERS images.

In Figure 6A, is reported a Raman image of a typical PNT2 cell after 2 h incubation; the color code of the map refers to pale-yellow as the highest intensity and dark-brown as the lowest. Figure 6B displays the superposition of the Raman image with the dark-field optical micrograph of the cell. SERS signaling is very limited and it is localized in a single spot within the cell. Most of the cell area is featureless, i.e., displays a flat baseline. This is a clear indication that the uptake is extremely limited. The amount of nanoparticles which are able to cross the cell membrane is exceedingly low. This same observation is verified for all of the investigated PNT2 population, and it is confirmed for longer uptake times (see Figure 7).

**Figure 8:**
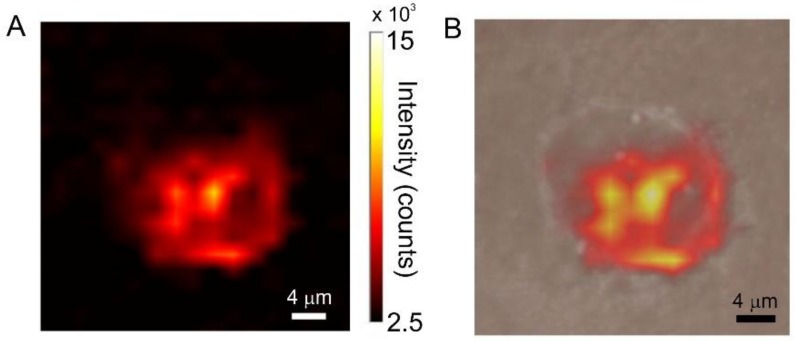
(**A**) SERS intensity map of a PC3 cell incubated for 2 h with AuNPs/TP. (**B**) superposition of the visible and SERS images.

**Figure 9:**
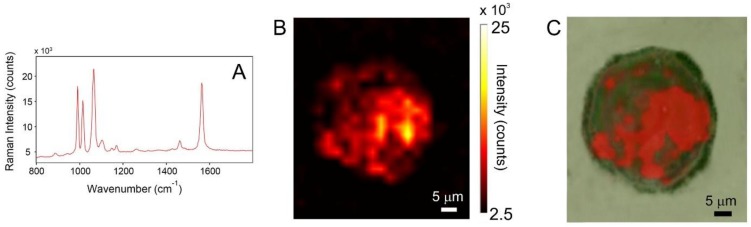
(**A**) SERS spectrum collected at the point of maximum SERS activity (bright yellow point in the Raman image). (**B**) SERS intensity map of a PC3 cell incubated for 6 h with AuNPs/TP. (**C**) superposition of the visible and SERS images.

**Figure 10:**
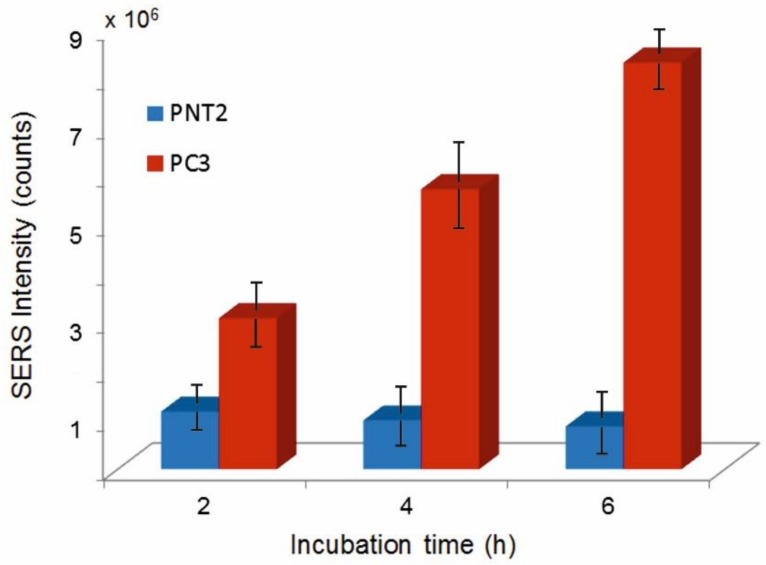
*I_1078_* vs. incubation time for PNT2 and PC3 cells.

A completely different situation is observed for the PC3 cell line. In this case, strong SERS signaling is already observed after two hours incubation (see Figure 8). The incubation time has a marked effect on SERS activity. This is demonstrated in the Raman/visible images of Figure 9 and in the bar-graph of Figure 10, representing *I_1078_* vs. incubation time for the two investigated cell lines. After 2 h incubation, SERS signaling in tumor cells is 260% higher than in normal cells. For PNT2, the uptake stops after 2 h while continuing at a substantial rate in the tumor cells. After 6 h incubation, *I_1078_* in the tumor cells is 950% higher than in the normal cells.

The Raman images show that in the PC3 line SERS activity is more or less homogeneously distributed in the whole cell area, rather than being localized in a single or in a few spots as for the case of the healthy PNT2 cells. In principle, the conspicuous enhancement of SERS activity in tumor cells could originate from different mechanisms. In particular, particle aggregation in the cellular environment cannot be entirely ruled out. However, the homogeneous distribution of SERS signaling in the PC3 line, with an average intensity that is close to that found at isolated spots in the healthy cells (compare Figure 8 and Figure 9 with Figure 6 and Figure 7) suggests that the effect is not due to the localized aggregation of nanospheres to form a limited number of hot-spots, which would produce spatially confined features with much more intense signals. Instead, Raman imaging suggests that the nanoparticles are evenly dispersed in the tumor cells and produce SERS signals comparable to those in the healthy cells.

The results discussed so far demonstrate the significantly higher uptake of AuNPs/TP by the PC3 line and that the functionalized AuNPs can be conveniently used for developing *in-vitro* methods to quickly and consistently differentiate healthy and malignant prostatic cells. The AuNPs/TP selectivity might also have therapeutic implications for targeted drug-delivery. The present results also highlight the sensitivity and specificity of SERS-based imaging for cytotoxicity studies.

## 4. Conclusions

Gold nanospheres of uniform shape and controlled size and size-distribution have been prepared to be used as *in-vitro* SERS probes for cellular uptake and localization. These nanoparticles have been subsequently functionalized with thiophenol and incubated in human prostatic cells with the aim of developing a robust, SERS-based method to differentiate normal and tumor cell lines.

The native AuNPs and the TP-functionalized AuNPs have been fully characterized by TEM, image analysis, DLS, UV, and Raman spectroscopy. No evidence of nanoparticle clustering was found, which is important for the present application, since the internalization process strongly depends on particle size. Cellular uptake has been monitored as a function of incubation time by SERS imaging, which demonstrated that tumor cells are able to uptake a significantly larger amount of nanoparticles in comparison to healthy cells (up to 950% larger after 6 h incubation). Relevant differences were also found in the uptake kinetics, which stopped after 2 h for healthy cells, while progressing at a substantial rate for the tumor cell in the investigated time-interval (0–6 h). This largely different behavior may result in being useful for diagnostic as well as therapeutic applications. This study also demonstrates that the intense and specific SERS signals of AuNPs/TP make them sensitive, robust, and low-cost probes for cellular uptake studies.

## Figures and Tables

**Table 1:** Assignments and symmetry of the principal normal modes in the 900–1600 cm^−1^ range for the Raman and Surface Enhanced Raman Spectroscopy (SERS) spectra of thiophenol (TP).

Raman (cm^−1^)	SERS (cm^−1^)	Symmetry	Assignments [29]
920	─	─	S─H in-plane bending
1003	1002	a_1_	Ring out-of-plane deformation/C–H out-of-plane bending
1028	1027	a_1_	Ring in-plane deformation/C–C symmetric stretching
1096	1078	b_1_	C–C anti-symmetric stretching
1122	1117	─	─
1161	1159	b_1_	C–H in-plane bending
1184	1182	a_1_	C–H in-plane bending
1587	1576	b_1_	C–C symmetric stretching

## Supplementary Materials

The following are available online at http://www.mdpi.com/2079-6374/8/4/87/s1, Figure S1: DLS analysis of the AuNPs colloid. Figure S2: Absorbance vs concentration plot for AuNPs colloids. The inset displays the UV spectra of the colloids. Figure S3: Comparison between the UV spectra of the AuNPS and the AuNPs/TP colloidal solutions. Absorbance values are normalized by molar concentration of nanoparticles. Figure S4: DFT optimized molecular structure of thiophenol, displaying the Van der Waals surface and the elliptic equatorial cross-section.
