# Supplementary material for: Functionalized Gold Nanoparticles as Biosensors for Monitoring Cellular Uptake and Localization in Normal and Tumor Prostatic Cells
Source: Biosensors (Basel). 2018 Oct 4;8(4):87. doi: 10.3390/bios8040087 (PMC6316160; doi:10.3390/bios8040087)
Supplement: Supplementary file 1 [file biosensors-08-00087-s001.pdf]

Functionalized Gold Nanoparticles as Biosensors for Monitoring Cellular Uptake and Localization in Normal and Tumor Prostatic Cells

Marianna Pannico[1,*], Anna Calarco[2], Gianfranco Peluso[2], Pellegrino Musto[1,*]

[1] National Research Council of Italy, Institute for Polymers, Composites and Biomaterials, 80078 Pozzuoli (NA), Italy.

[2] National Research Council of Italy, Institute of Agro-environmental and Forest Biology, 80131 Naples (NA), Italy.

Supplementary Data

Table of contents

DLS measurements pag.2

UV Spectroscopy pag 2

Evaluation of the equatorial cross-section of thiophenol pag.3

1. DLS Measurements

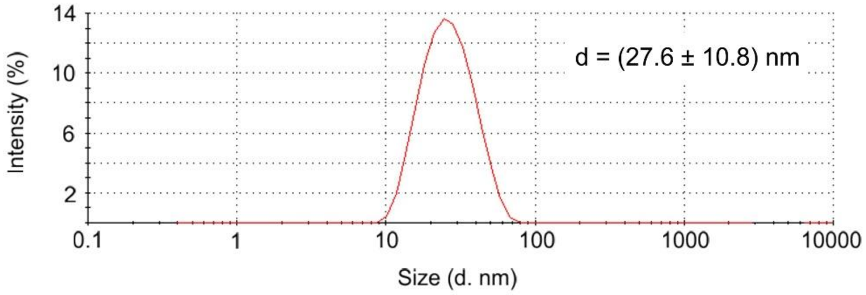

Figure S1. DLS analysis of the AuNPs colloid.

2. UV Spectroscopy

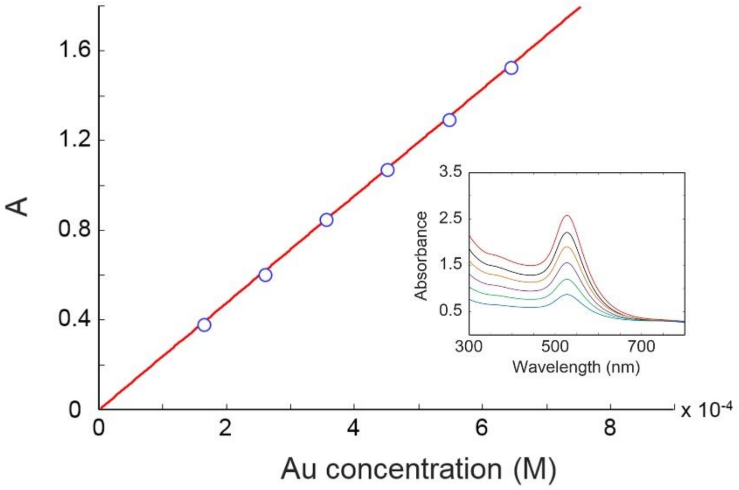

Figure S2. Absorbance vs concentration plot for AuNPs colloids. The inset displays the UV spectra of the colloids.

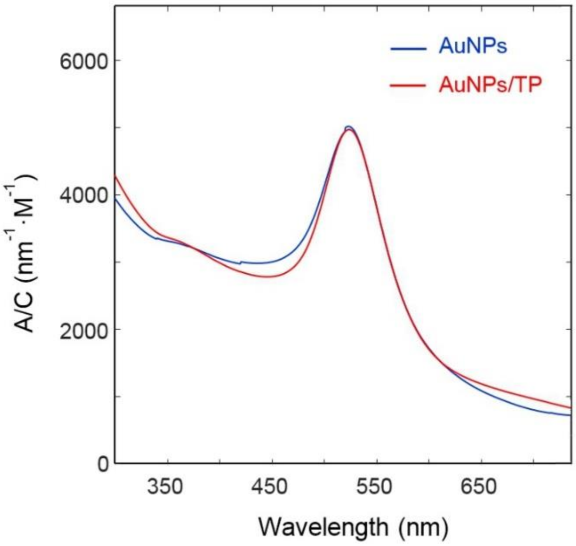

Figure S3. Comparison between the UV spectra of the AuNPS and the AuNPs/TP colloidal solutions. Absorbance values are normalized by molar concentration of nanoparticles.

3. Evaluation of the equatorial cross-section of thiophenol

The isolated thiophenol molecule was preliminary optimized at DFT/B3LYP level of theory using the standard 6-31G(d) basis set. The Van der Waals surface was then visualized and the two semi-axes, *a* and *b,* of the equatorial ellipse were measured. The molecular model, along with the relevant geometric parameters, is represented in Fig. S4.

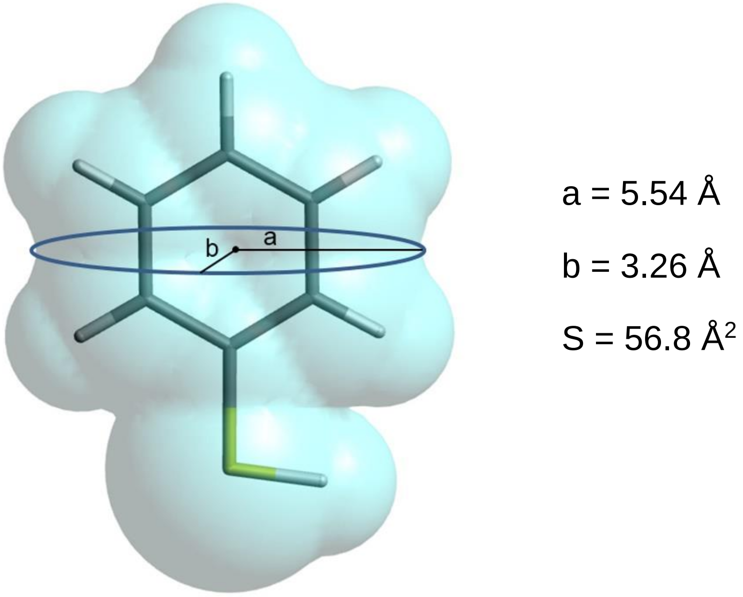

Figure S4. DFT optimized molecular structure of thiophenol, displaying the Van der Waals surface and the elliptic equatorial cross-section.
